# Observations and Essays on the Statistics of Insanity; Including an Inquiry into the Causes Influencing the Results of Treatment in Establishments for the Insane: To Which Are Added the Statistics of the Retreat, near York

**Published:** 1846-10

**Authors:** 


### Art. VI.

*Observations and Essays on the Statistics of Insanity; including an Inquiry into the Causes influencing the Results of Treatment in Establishments for the Insane: to which are added, the Statistics of the Retreat, near York.* By John Thurnam, Licentiate of the Royal College of Physicians of London, Resident Medical Superintendent of the Retreat, near York.—*London,* 1845. 8vo, pp. 308.

In the year 1841, a report, by Dr. Thurnam, including a series of statistical tables, exhibiting the practice of the Retreat, the asylum for the insane of the Society of Friends, for a period of 44 years, from its first establishment in 1796 to 1840, was printed for distribution by the directors of the institution. Of this valuable report a brief notice was inserted in our Journal at the period. The work now before us contains an enlarged and revised edition of the "Statistics of the Retreat," preceded by an elaborate investigation of the present state of the statistics of insanity, deduced from reports and other data furnished by hospitals for the insane in this country, on the Continent, and in America. This portion of the work, we are informed by the author, had its origin at the time he was engaged in drawing up the "Statistics of the Retreat," in the desire to compare the results afforded by the practice of that institution with those obtained in other asylums and hospitals for the insane.

The subjects treated of by Dr. Thurnam are arranged under three heads, the discussion of which is preceded by a concise exposition of the value of statistics as applied to the elucidation of insanity.

I. On the Statistics of Insanity in general. This may be regarded as constituting the body of the work, and is distributed in three chapters, which are devoted—1st, to the methods of deducing and exhibiting the results of treatment in institutions for the insane; 2d, to the various circumstances capable of influencing these results; and 3d, to the statistics of the principal asylums of this and other countries.

II. Essays on the liability to insanity in the two sexes, at different periods of life, and in the Society of Friends.

III. The Statistics of the Retreat, preceded by a sketch of its history and an exposition of its system of management, and of the methods of treatment pursued. To these are added, in an appendix, the history and statistics of the York Lunatic Asylum, and contributions to the statistics of the Society of Friends.

### I. On the Statistics of Insanity in General.

1. In order accurately to exhibit the results of the practice of different establishments for the treatment of insanity, it is necessary that the terms employed should be clearly defined; that the registers should be correctly kept; that the mode of calculating the proportion of recoveries and deaths should be uniform; and that the data for calculation should be deduced from observations extending over a sufficient period of time. To the first requisites it is not necessary further to allude: in reference to the calculation of the proportion of recoveries and deaths, various methods have, however, been adopted in different institutions, and the periods of observation have often been too limited to allow of any precise comparison.

Dr. Thurnam, following the plan adopted by Mr. Farr, in his pamphlet on the "Statistics of English Lunatic Asylums," published about ten years ago, has calculated the proportion of recoveries on the number of cases admitted. To this plan it has, however, been objected, that it exhibits results less favorable than the true proportion, as of the whole of the cases, some still remaining under treatment may admit of cure, and yet have no account taken of them. Mr. Farr, accordingly, in his paper on the "Mortality of Lunatics," in the Statistical Journal for 1841, adopted the method of calculating the proportion of recoveries on the numbers discharged, and in the Reports of the Glasgow Asylum and those of the State Asylum at Worcester, Massachusetts, U. S., the same plan has been followed. This mode, we think with Dr. Thurnam, is still less accurate than the former, the results being much too favorable; as, of the cases received into any institution, a considerable number remain in a chronic state, to swell, at a later period, its mortality. Mr. Farr has, indeed, met this objection by stating that, "if the mortality remained the same, the probability is that the patients to be discharged would, *cæteris paribus*, be discharged cured, relieved, and died, in the same proportion as those already discharged." Our author, however, shows that the results of observations do not confirm Mr. Farr's theoretic statement—of the patients remaining in an institution at any period, a much less number being in a curable state than its average proportion of cures. Thus, at the Retreat, at Midsummer 1840, the proportion of cases regarded as curable was only 15 per cent., while the average proportion of recoveries during the 44 years that it had been in operation, amounted to nearly 47 per cent.

Under either of these methods the results are not strictly accurate, the true proportions being intermediate between those thus obtained. The former plan, however, probably possesses the greatest exactitude, and this view is confirmed by the fact, that while, according to the latter method, the proportion of recoveries at any institution is in its early periods high, and undergoes a rapid diminution; that furnished by the former gradually increases at each quinquennial period for a long series of years.

The most accurate method of estimating the average mortality of hospitals for the insane is, doubtless, as shown by Mr. Farr, to calculate the proportion of deaths on the average annual population or mean number of patients resident in the institution. In the reports of some asylums, however, the mortality has been estimated by the proportion of deaths to the patients admitted, and in others, to those discharged, while in some, amongst the most recent of which may be mentioned that of M. Parchappe, on the asylum at Rouen, the error has been committed of calculating the mortality on the total number resident during each year. Our author shows that our judgment of the character of these establishments, as founded on the rate of their mortality, will be entirely reversed according as the proportion of deaths is calculated on one or other of these plans, and, we need scarcely observe, that, without the employment of uniform methods of estimating the mortality, no satisfactory comparisons can be instituted.

In estimating the relative mortality of acute diseases, which have generally a definite course and duration, the period of residence is of little importance, but in an affection so chronic as insanity, and of which the duration is so varied, no comparison can be made between the average mortality of different institutions, if time be not taken into the account. Dr. Thurnam, therefore, justly expresses regret that in so few of the reports of asylums has the average population, or mean number of patients resident in the institution been hitherto given. We would further observe, that the same omission exists in the reports of many of our general hospitals, in which, though of little importance as applied to estimates of the relative mortality of similar acute diseases, the average population is a necessary item in all comparisons of general results, and calculations of an economical nature.

To ensure accuracy in the comparison of the mortality of lunatic asylums, it is also necessary that the estimate should be founded, not merely on the total number of patients resident, but on the numbers at the several periods of life respectively:—since it is evident that the varying mortality at different ages may materially affect the general results in institutions receiving different proportions of patients at the several periods of life.

All who are familiar with statistical inquiries are aware of the fallacious results of calculations deduced from limited series of facts and short periods of observation; the period, however, over which it is necessary that the observations should extend, in order to ensure accuracy in stating numerically the results of the practice of lunatic asylums, appears, from our author's researches, to be greater than would *a priori* have been anticipated. At the Retreat, he informs us, that the period of twenty years elapsed from the establishment of the institution, before the proportion of cures arrived at the point which it has since maintained during the entire period of its operation. The rate of mortality during the first five years amounted to 5·71 per cent., and gradually declined from that period, till, at the end of 25 years, it averaged only 3·71 per cent.;—it then again underwent an increase; and, after the lapse of 35 years, attained the rate of 4·71 per cent., which it has since preserved. From an investigation of the statistics of other institutions, Dr. Thurnam has also found that the proportion of recoveries has, in nearly every instance, gone on increasing for a period often of 30 to 40 years from their commencement, owing probably, to the large number of chronic cases admitted at the first opening of any asylum, and to the long period occasionally required for the cure of the disease. On the other hand, it appears that the mortality is generally more favorable in the early periods of the operation of an asylum, and gradually increases for 20 or 30 years, when, as more recent cases are admitted, and the older ones die off, it often presents a rate of 50 or 100 per cent. higher than that of the first few years. Dr. Thurnam, therefore, suggests that, in the published reports of asylums, a summary should be appended of the general results for a series of years, dating from the establishment of the institution, or, if any great change have taken place in its mode of management, from the period of such change.

2. In treating of the circumstances in the character of the cases admitted and the method of treatment pursued, as influencing the results obtained iu hospitals for the insane, Dr. Thurnam adopts with some modification the arrangement of Gavarret, into—1st, individual conditions; 2d, hygienic conditions anterior to the invasion of the disease; 3d, the form of disease, its duration, &c.; 4th, the hygienic conditions during treatment; and 5th, the treatment itself.

Of the individual conditions and the hygienic conditions anterior to the invasion of the disease, the chief circumstances which modify the results of treatment are the sex and age, rank and previous habits of the patients, and the predisposing and exciting causes of the disease.

*Sex.* Writers have differed as to the relative frequency of recoveries from insanity in the two sexes. The generally received opinion has however been, that in females the probability of recovery is not only greater, but that the tendency to fatality is less. With these views Dr. Thurnam's conclusions coincide. He states that, with two exceptions, in all the institutions the statistics of which he has examined, and of which the periods of operation have been sufficiently extended, the proportion of recoveries in females has exceeded, often to a considerable extent, that in men. The excess in favour of females he finds to vary in different institutions, from 4, 5, and 7 per cent. in the asylums of Glasgow, Belfast, and Lancaster; to 20, 22, 23, and 28 per cent. at the Retreat, the asylum of Schleswig, in Holstein, Charenton, and the York Lunatic Asylum. At the Senavra hospital, in Milan, the proportion of recoveries in females has only exceeded that in males by 2 per cent., and at Hanwell and the Bloomingdale Asylum, in the State of New York, U. S., the excess of recoveries is on the side of males, and amounts to 5 and 28 per cent. respectively. The results afforded by the experience of the Senavra may probably, it is suggested, be referrible to the cases treated in that institution being most generally complicated with the peculiar endemic of Lombardy—the pellagra. The great excess of recoveries in males at the Bloomingdale asylum is ascribed to persons labouring under delirium tremens being treated in that institution.

The difference between the mortality of insanity in the two sexes is greater than in the relative probability of recovery. Instead of the excess of 5 or 6 per cent., which obtains in the general population, our author's investigations show that, with the solitary exception of that of Belfast, the average mortality among males in the British asylums is uniformly very much greater than among females. At the York Lunatic Asylum and St. Luke's there has been an excess on the side of the men of 93 and 98 per cent. respectively; and, in the institutions where the difference is least, as in the asylums of Schleswig and Worcester, U.S., the mortality in males is 9 and 13 per cent. greater than in females. This difference applies to institutions admitting all ranks of patients in a nearly equal degree; being in the metropolitan licensed asylums for paupers 63 per cent.; and in those for private patients 57 per cent. At the Senavra, the mortality in females, during a period of 25 years, exceeded that in males by 13 per cent. This statement is sufficient to show the necessity for giving the average mortality of any institution in each sex separately, as it is evident that, by the respective proportion of males and females, the general results will be materially modified.

As regards the liability to relapses and recurrences of the disorder, the influence of sex would appear to be partially reversed, females being somewhat more frequently the subjects of second attacks than men. The results obtained by our author on this point, as also on the frequency of second attacks of insanity, being highly interesting, and opposed to the views generally entertained, we shall lay before our readers. The method of ascertaining the frequency of the recurrence of insanity which has ordinarily been adopted, has been to calculate the proportion which the cases readmitted into any institution, bear to the whole of those treated or discharged cured. Esquirol was thus led to estimate the relapses at 10, Pinel and Despertes at 17, and Farr at 30 per cent. of the recoveries. It is, however, obvious that a calculation of this kind is open to the fallacies that cases may be readmitted which were not recovered at the time of discharge, and that cases discharged cured may relapse, but not be again sent to the institution. At the Retreat the readmission of cases discharged recovered have amounted to 31·5 per cent. This must, however, our author remarks, be regarded as less than the true proportion of relapses ; as, of those discharged, some may yet, after several years have elapsed, experience second attacks. In support of which, he states that five men, labouring under second attacks, had been admitted since the date of the report, and after 4, 5, 8, 14, and 19 years had respectively intervened since the period of their recovery from the first attack. A further correction must be made for those who, having been discharged from the institution cured, were known to have experienced subsequent attacks, but had not been readmitted. Dr. Thurnam, therefore, infers that, by this method of investigation, "the liability to relapse or recurrence of insanity, after recovery from a first attack, all things considered, can scarcely be estimated as at all less than 50 per cent., or as 1 in every two cases discharged recovered."

As stated by Mr. Farr, it is, however, only by following through life a large series of cases where recovery has occurred, that the frequency of second attacks of insanity can be satisfactorily ascertained. The Retreat affords peculiar facilities for such an investigation ; as "not only is almost every case of second attack readmitted, but an opportunity may generally be found for ascertaining the subsequent history of all cases that have at any time been under care." Dr. Thurnam has thus been enabled to obtain full information as to the whole of the 244 persons who have at any time been patients in the institution, and who have subsequently died. Of these it appears that only 131, or 53·6 per cent. recovered from the first attack ; and of these 45, or nearly one third (18·4 per cent. of the whole) continued permanently free from mental disorder, while 86 had one or more subsequent attacks, and only 20 were sane at the time of death. Of the 244 cases, therefore, only 65 (45 + 20), or 26·6 per cent. died sane.

"In round numbers, then, of 10 persons attacked by insanity, 5 recover and 5 die, sooner or later, during the attack. Of the 5 who recover, not more than 2 remain well during the rest of their lives ; the other 3 sustain subsequent attacks, during which at least 2 of them die. But although the picture is thus an unfavorable one, it is very far from justifying the popular prejudice, that insanity is virtually an incurable disease ; and the view which it presents is much modified by the long periods which often occur between the attacks, during which intervals of mental health (in many cases of from 10 to 20 years' duration) the individual has lived in all the enjoyments of social life."

Our author's observations show that the proportion of recurrences in women is somewhat greater than in men; being, in the former, 67·1, in the latter, 65·7 per cent.

*Age.* The greater curability of insanity in early life has been long established. Esquirol regarded the disease as most amenable to treatment in persons between 20 and 30 years of age. This result differs somewhat from the conclusions of our author; who, from the tables of the Retreat, York Asylum, and Bethlem Hospital, infers that the recoveries are most numerous at the earliest ages at which the disease prevails, and gradually decrease in number with the progress of life. The proportion of the recoveries under 20 years of age being 55·55 per cent. of the whole of those admitted between these ages; 51·77 per cent. between 20 and 30 years; 46·16 between 40 and 60; and 27·8 between 60 and 80. The decrease in the proportion of recoveries from 40 to 60 and 80 years of age, follows, therefore, a rapidly increasing ratio, being 7, 11, and 40 per cent. respectively. The only exception to the increasing proportion of recoveries with the progress of age, is stated to exist in females; in whom the proportion of recoveries at 10 and 20, and 40 and 50, is less than in the subsequent periods of 20 and 30, and 50 and 60 years of age; exceptions of which the peculiarities of the sex afford a probable explanation. The mortality of the insane at different periods of life has been made the subject of an elaborate investigation by our author; and the table which he has compiled from the experience of the Retreat and York Lunatic Asylum, constitutes, we believe, the only approach to a life-table for the insane which has yet been published. The rate of mortality, as observed at the Retreat, has been adopted by the Medical Invalid Life Assurance Society, for the calculation of the rates of insurance on the lives of insane persons under favorable circumstances. From the table showing the estimated mean annual mortality at these institutions, as compared with that in the population at large, and in the Society of Friends, it appears that, supposing 100 persons were living at each decennial period of life, from 20 to 90 years of age, instead of the mean mortality at all ages being, as estimated by Edmonds, for the country at large, 6·03 per cent., or as deduced from the data given in the Appendix for the Society of Friends alone, 4·8 per cent.—the mortality of the York Lunatic Asylum is found to be 10·88, and that of the Retreat 7·32 per cent. While, however, there thus exists a great excess in the mortality of insane persons at all ages over that of the population at large, this excess is by no means equal at each period of life, being much greater at the early ages than in more advanced life. Dr. Thurnam remarks that—

"The mortality at the Retreat, especially among males, from 20 to 50 years of age, was double, treble, and even quadruple, what it is among the sane at the same ages; whilst at 70 years of age and upwards, it is only slightly, if at all, higher than in the population at large. And thus it appears not improbable that if the insane survive the 60th year, they are nearly as likely to attain advanced age as the sane. At this period of life, the disorder has in general passed into a fatuity, or, at least, into a chronic and often lively form of insanity; and the bodily functions are often remarkably active, if not also vigorous. The prevalent error of the insane being long-lived is, perhaps, to a certain extent, to be explained by this interesting and unexpected result."

The influence of insanity on the duration of life is further illustrated by tables giving the age of the patients, the duration of the disorder, and the period of death, in the whole of the cases which have proved fatal at the Retreat since its establishment. These cases are 139 in number, and the average age at which such of the patients as were members of the Society of Friends were attacked with the disorder was 39·19 years; of the others, 38·6 years; and the mean age at the period of death was 56·5 and 47·7 respectively. In reference to these results, our author remarks that, as at 38 and 39 years of age, the expectation of life is regarded as not less than 28 years, the mean ages attained should have been 67 and 66, instead of 56 and 47 respectively. "In those connected with the Society, therefore, less than two thirds, and in others not more than a third, the expectation of life at the time of attack was, on an average, realized." Notwithstanding the occasional occurrence of instances of longevity amongst insane persons labouring under the chronic form of the disease, the tendency of insanity to shorten life is thus shown to be very decided, owing to the high rate of mortality in the earlier stages and more acute forms of the disease. It is further stated, that in those who recovered from first attacks, and died subsequent to discharge, the ages at the time of death and at the period of attack corresponded with those observed in the institution; evincing that after recovery from insanity, as after attacks of most important diseases, the average duration of life is materially shortened.

3. Few subjects have recently attracted more deserved notice than the influence of distress and destitution, and their attendant evils, in increasing the prevalence of disease, and lessening the average duration of life; and it was to be anticipated that the experience of asylums for the pauper population, more especially of the large commercial and manufacturing towns, would present results less favorable, both as regards the recoveries and mortality, than institutions wholly or in part devoted to the reception of persons of the more opulent classes. This inference is confirmed by our author's researches; and he further thinks it probable "that the proportional difference in the rate of mortality in different classes of society is much increased under the operation of insanity, and that it is greater than under that of disease in general." In support of this opinion he contrasts the proportion of recoveries and mortality at the Retreat with their rate at the asylums of Wakefield and Lancaster, for the paupers of the West Riding of Yorkshire and Lancashire. In the former institution the proportion of recoveries, in persons connected with the Society of Friends, has been about 50 per cent., and the mean mortality 4·7 per cent.; whilst at Wakefield and Lancaster the recoveries have only been 43·6 and 41·4 per cent., and the mean mortality has attained the high averages of 15·7 and 16·5 per cent. respectively. A similar, though less marked, difference is observed in the metropolitan licensed private asylums; the mortality having been 20·68 per cent. in such of the houses as receive paupers, whilst in those for private patients it was only 10-94 per cent. In reference to the remarkable contrast thus exhibited, Dr. Thurnam remarks that it can scarcely be entirely attributed to the previous circumstances of the patients, but must, in part at least, be referred to the pauper patients not enjoying an equal degree of domestic comfort and as nutritious a diet as the rich.

Passing over the fourth and fifth classes of circumstances modifying the results of treatment in asylums for the insane, we shall give our author's conclusions as to the influence exerted on the statistics of such institutions, by the duration of the disorder at the period of admission, and by the duration of residence or treatment.

The duration of the attack at the time of admission into hospitals for the insane exerts a double influence on the statistical results;—the probability of recovery being much greater in such cases as are brought under care at an early period, while the average mortality is less in the chronic stages of the disease.

"At the Retreat, the probability of recovery in cases brought under care within three months of the first attack, has been found to be as 4 to 1, and excluding cases complicated with serious bodily disorders, as 9 to 1; whilst in cases not admitted till more than twelve months after the attack, the probability of recovery is less than as 1 to 4. The proportion of recoveries now mentioned as having occurred at the Retreat, in uncomplicated cases of less than three months' duration, or that of 9 out of every 10 cases brought under care, is the same as that which, in cases equally recent, the late Dr. Willis and Dr. Burrows both stated as having occurred in their private practice. Though, both when first made, as well as since, Dr. Willis's statement has often been called in question, it may still be regarded as not in any degree improbable, and may, indeed, often apply to a small number of cases. But unless a selection be made of uncomplicated cases, it may be questioned whether so large a proportion will be found to recover when the experience is not a limited one. Much reliance, indeed, should never be placed upon the results derived from a limited sphere of observation, whether in public or private practice, as proof of which it may be here stated, that out of twenty cases admitted at the Retreat within three months of the first attack, during 10 years (1798-1808), 19 were discharged recovered."

As before stated, the short duration of the disorder on admission exerts an unfavorable influence on the rate of mortality. During 48 years, the annual mortality at the Retreat averaged 7·3 per cent. in cases of first attack admitted within three months of the commencement of the disorder, and only 4·7 per cent. in cases whether of first attack or otherwise, and of more than twelve months' duration. These differences in the results of treatment in cases of insanity, according to the period at which they are admitted into hospital, show that to enable any satisfactory comparisons to be made between the results of different institutions, it is necessary that similar modes of classification of the cases as to duration at the time of admission should be adopted. The cases at the Retreat are arranged into four classes—1st, cases of the first attack, if not more than three months' duration at the time of admission; 2dly, cases of the first attack of more than three, but not more than twelve months' duration; 3dly, cases not of the first attack, and of not more than twelve months' duration; and, 4thly, cases whether of the first attack or not, and of more than twelve months' duration. This plan, or one almost identical with it, our author informs us, has already been introduced into several of the county asylums, and he suggests that a similar classification should be generally followed.

The duration of the treatment, or residence, may be modified by a variety of causes; as by the results of treatment being more or less favorable, and so having a smaller, or larger, proportion of cases in an incurable condition; by economical motives occasioning an early removal of the patients from the institution; or by special rules limiting the duration of their residence. At the York Lunatic Asylum, where the second cause operates, the patients, on an average, only remain 2½ years (2·52); at Bethlem and St. Luke's, where they are only permitted to remain twelve months, the duration of residence of curable cases averages only half a year (·57), and two thirds (·68) of a year respectively. While at the Retreat, where, from the peculiar constitution of the Society of Friends, neither economical nor other motives operate to hasten removal, the mean period of residence has been nearly five years (4·8). In the latter institution one third (34 per cent.) of the entire recoveries have occurred after the first year of residence; and nearly one sixth (or 15·3 per cent.) after the second year. Though, therefore, at the Retreat, in some of the cases the period of probation may have been unnecessarily prolonged, it is evident that the short duration of residence at the former institutions must operate unfavorably on their proportion of recoveries, and, by occasioning the premature discharge of cases, must lessen the probability of ultimate recovery. The long duration of residence at the Retreat not only proves favorable to its proportion of cures, but lessens the average mortality.

The influence exerted on the statistics of hospitals for the insane by the several particulars of treatment, is treated of under the two heads of the general hygienic conditions during treatment, and the treatment itself. Of the former class of modifying causes are—1st, the healthiness of the locality in which the asylum is situated, as influenced by climate, elevation, soil, drainage; 2d, general adaptation and appropriate construction of the buildings; 3d, means of exercise, occupation, and amusement; 4th, internal economy and government, number of attendants; 5th, ventilation, lighting, warmth, and cleanliness of the apartments, as modified by the numbers treated in the institution; 6th, clothing and personal cleanliness, baths, &c.; 7th, diet. Of these various circumstances our author takes a rapid survey, and weighs the probable effect of their good or bad arrangement on the proportion of recoveries and mean annual mortality of institutions for the insane. In reference to the internal economy and government of asylums, he agrees in the opinion, which we believe to be general at the present day, that the most efficient system of management is that in which the entire direction is vested in a resident medical officer, assisted by the advice of visiting physicians. Such a system, to be thoroughly carried out, presupposes the limitation of asylums to the accommodation of a moderate number of patients. Dr. Thurnam quotes on this subject the opinion of Jacobi, that the maximum number of patients to be admitted into one asylum ought never to exceed 200; probably, however, in pauper asylums, accommodation for 300 or 400 patients is not too great. Our author also expresses his approbation of the suggestion to erect *asylums* for the detention and care of the incurable and harmless patients, apart from the *hospitals* for the treatment of the more acute and severe cases of insanity; and suggests that such institutions should be in the immediate neighbourhood of the latter, and under the charge of their medical officers. We believe that this change, more perhaps than any other, would conduce to further the efficiency of our larger public asylums; many of which are at present so crowded with patients in a state of fatuity and in the chronic forms of the disease, as to limit the accommodation for the more acute and urgent cases, and interfere materially with their successful treatment.

The number of attendants on the patients in institutions for the insane is of great importance, as affecting not only the comfort of those under treatment, but influencing a variety of circumstances by which the general efficiency of an establishment, and its consequent proportion of recoveries, and amount of bodily disease and mortality, are materially modified. The proportion which the attendants bear to the patients in different asylums seems to vary considerably. In 13 county asylums, in the year 1835, the average proportion was 1 attendant to 17 patients; and, in some of these establishments the number was much less. In the Wakefield Asylum there was only 1 attendant to 20 patients, and at Hanwell 1 to 26; but the proportion has since been increased in the latter institution to 1 to 18. In 8 of the 10 Irish district asylums there is 1 attendant and assistant to 9 patients; or, taking responsible attendants only, 1 to 13 patients. At the York Lunatic Asylum and the Lincoln Asylum, which receive both pauper patients and those of the upper ranks of society, the proportion of attendants is 1 to 11 or 12, and 1 to 9 patients. In the asylums of Montrose, Aberdeen, Glasgow, Dundee, and Perth, the proportion appears to average 1 to 10. At Siegberg, near Bonn, it is 1 to 7 or 8; and at the Retreat, 1 to 6 or 7 patients. Our author concludes the enumeration of the proportion which the attendants bear to the number of patients under treatment at different institutions by remarking that—

"In pauper *hospitals* for the insane, in which the patients are frequently able to render considerable assistance to the attendants, the proportion of the latter will not, perhaps, on the average, require to be more than 1 to 12 or 15; and in *asylums* for this class the proportion need not be half even this. In other institutions, the highest class of patients, on account of their previous habits, will often require a separate and distinct attendant to each case. This should, perhaps, always be the case in the early stages and more severe forms of the disorder; but in the more advanced stages, and more chronic and harmless forms of insanity, a limited number of this class, not exceeding five or six, may be properly enough associated together under the care of two or three attendants."

The diet of the patients in the various county asylums for paupers differs considerably, both in the quality and quantity of the food; and, in some, the unfavorable results of treatment, as evinced by the low proportion of recoveries and high rate of mortality, is probably in part ascribable to a deficiency in the amount of nutritious food allowed. With a view to illustrate this subject, our author has given a table of the diet in seven of the principal county asylums, as it existed in 1835. These institutions, he shows, may be divided into two groups; in one of which, including the asylums of Nottingham, Stafford, and Gloucester, the diet was considerably above the average of similar institutions; while, in the other group, embracing the four asylums of Wakefield, Lancaster, Suffolk, and Middlesex, the allowance of food was much below the average. In the first group, the solid food, consisting of meat, cheese, puddings, bread, and other articles, exclusive of vegetables, amounted to 225 oz. per week; while in the second it was only 150½ oz. The amount of animal food alone was, in the first group, 46 oz., and in the second only 19½ oz. per week; and, though the allowance of soup, porridge, milk, &c., was in the latter 15½ pints per week, while in the former it was only 10 pints, this execss could by no means compensate for the deficiency of more solid nutriment. In the first group, also, the quantity of beer allowed amounted to 2 pints daily; in the latter to not more than half a pint. The difference in the results as to recoveries and deaths between these two groups of institutions is very striking; in the three asylums with the more liberal diet, the recoveries having been 43·7 per cent., and the mean mortality 9·35 per cent.; while in the second group, with the more scanty allowance of nutritious food, the recoveries have averaged only 36·75 per cent., and the mortality has been as high as 14·54 per cent. It would be premature, on the limited information before us of the circumstances of these several institutions, to draw any positive inference as to the dependence of their comparatively successful treatment on the respective diets allowed; it seems, however, not improbable that a portion, at least, of the discrepancy may be referred to this cause. The observations call for a fuller investigation into the effect of diet on the treatment in pauper asylums; and it would be well that in these institutions, as in workhouses and prisons, fixed dietaries, apportioned on a liberal scale to the several classes of patients under treatment, and convertible so as to present varieties in the kind of food allowed, should be arranged by the commissioners, and required to be generally observed.

The remaining portion of this section of Dr. Thurnam's work is devoted to the consideration of the several objects to be held in view in the treatment of insanity, and the influence of the treatment pursued on the statistical results of hospitals for the insane. The section concludes with an illustration drawn from the history of the York Lunatic Asylum, prior and subsequent to its reformation in 1814. It is impossible to read this and other accounts of the state of our asylums a few years ago, without feelings of great gratification with the changes which have taken place. In reference to this institution, it is satisfactory to find that the abandonment of a system of management fraught with cruelty and abuse, and the adoption of plans of treatment more accordant with humane feelings, have been attended with a very decided improvement in the health of the inmates, as evinced by the decrease in the average mortality. Thus our author shows that, during the 37 years from the establishment of the institution to the period at which the change in the management took place, the average mortality was 11 per cent.; and during the six years which immediately preceded that change, and when the abuses in the asylum had attained their greatest height, not less than 14·8 per cent. Since this period, on the contrary, in the 26 years which elapsed to 1844, the mortality has been only 7·24 per cent., or 40 per cent. less than its rate during the entire former period, and fully 100 per cent. less than during the six years preceding the change. The mode in which the registers of the patients were kept before the reform precludes the possibility of any investigation of the relative proportion of recoveries; but there can be no doubt that the improvement in this respect has not been less satisfactory.

The third and concluding chapter of this portion of Dr. Thurnam's work contains the statistics of the principal hospitals for the insane in this country, on the Continent, and in America. An elaborate table presents us with the data furnished by the entire experience of 59 public asylums, including the observation of more than 125,700 cases of insanity; together with calculations of the proportion of recoveries in the numbers admitted, and the mean annual mortality estimated on the average population. We quote the following tabular statement of the results deduced from this laborious investigation:

| | Proportion of recoveries. | Mean mortality. |
|---|---|---|
| 9 English county asylums, receiving paupers . . . | 36·95 | 13·88 |
| 6 English county asylums, receiving paupers and private patients . | 46·87 | 10·46 |
| 8 English asylums, supported by charitable contributions, and receiving pauper and private patients . . . . | 40·94 | 8·93 |
| 7 Scotch asylums, receiving pauper and private patients . . | 42·37 | 7·52 |
| 10 Irish district asylums for paupers . . . . | 48·33 | 8·7 |
| 5 asylums in the United States for paupers and others . . | 46·82 | 9·56 |

From this table it is inferred that, in asylums which have been established for a considerable length of time, the proportion of the recoveries to the whole of the cases admitted should be considered low, if amounting to much less than 40 per cent., and high if exceeding 45 per cent. On the other hand, the mortality in an asylum for the more opulent classes of society, as well as for paupers, calculated on the average numbers resident, may be considered highly favorable if under 7 per cent., and decidedly unfavorable if exceeding 9 or 10 per cent. In asylums wholly appropriated to paupers, a mortality of much less than 10 per cent. must be regarded as very low, and one exceeding 12 or 13 per cent. as decidedly high. While, however, the sources of fallacy in estimating the relative efficiency of different institutions by the average mortality in the resident population are fewer than those which attend conclusions drawn from the proportion of recoveries, great caution must be exercised in adopting either of these standards of comparison.

### II. ESSAYS ON THE LIABILITY TO INSANITY.

1. *Relative liability to insanity in the two sexes.* It has been the generally received opinion since the time of Aretæus and Cælius Aurelianus, that females are more subject to insanity than males, and this Esquirol, from an extensive statistical investigation, believed to be correct, inferring that 38 females became insane to 37 males; a view which has since been maintained by most writers on insanity. In estimating, however, the relative liability of the two sexes to this disease, Esquirol took no account of the relative proportion of the sexes living at the periods of life when insanity is most frequent; an omission which, with the known preponderance of females in the general population above 15 and 20 years of age, goes far to explain the results he obtained.

In this country Dr. Thurnam states that the only institutions which maintain a much larger proportion of females than males are those in the neighbourhood of the metropolis, Bethlem, St. Luke's, and Hanwell; and in the latter there has been of late years a gradual diminution of the excess in females. Their greater proportion in these asylums may perhaps be explained by the preponderance of females being still greater in the population of London than in that of the country at large, amounting in the former to 118 females to 100 males from 20 to 50 years of age, while in the latter there are only 108 females to 100 males. The greater proportion of insane females in these institutions may also be in part referrible to peculiar circumstances connected with the metropolitan population. The excess seems only to exist in the inferior ranks of society, as in the licensed metropolitan asylums for private patients, males are in the largest proportion.

Esquirol also, in his statistical investigation on this subject, estimated the relative proportion of the two sexes in the *existing*, not in the *occurring*, cases of insanity; a method of inquiry which, when the relative mortality of the disease in the two sexes is considered, is obviously fallacious. Dr. Thurnam has shown that, while in the general population of this country the mortality of adult males exceeds that of females only by 7 or 8 per cent., the excess of mortality in insane persons of the male sex is not less than 50 per cent., and consequently that the gradual accumulation of incurable females in our asylums is, in this proportion, greater than that of males. It is obvious, therefore, that the question of the relative liability of the two sexes to mental disease must be decided by the relative proportion of occurring cases in each; or, as the only available means of ascertaining this, by the relative numbers admitted during a term of years into our hospitals and asylums for the insane. With this view, Dr. Thurnam has compiled a table of 48,103 cases admitted into 32 different asylums in this country, America, and on the Continent, from which it appears that in 24 of these institutions the admissions of males were the most numerous, the excess in the whole number amounting to 13·7 per cent., and in some of the English institutions being not less than 25, 30, and 40 per cent. The only English county asylums where this excess does not exist are those of Dorset and Norfolk. From a still larger number of cases, including persons of all ranks of society, he elicits similar results; and it would appear from the greater excess of males admitted into the asylums of the metropolitan district for private patients, that in the upper ranks of society men are more liable to insanity than women, in a still higher proportion than obtains in the pauper population. It must, however, be borne in mind, that among the wealthier classes a larger proportion of insane females may possibly be detained under the charge of their friends.

The experience of the Society of Friends, as ascertained by the admissions into the Retreat, would appear at first sight opposed to these conclusions; the excess of females admitted since its commencement amounting to 18 per cent. It is, however, probable, that amongst adults the excess of females in this community exceeds that of males in a larger proportion than in the general population, being, our author infers from data with which he presents us in an appendix, not less than 30 to 35 per cent.

In most respects, therefore, as regards insanity, females are more favorably circumstanced than males. They are less liable to the disease; and when seized, the prospect of their recovery is greater, and the tendency to death less; they are, however, apparently somewhat more exposed to relapses and recurrences of the disorder.

2. *On the relative liability to insanity at different ages.* Esquirol, proceeding on the same erroneous method of statistical analysis, as in estimating the comparative frequency of insanity in the sexes, inferred that the tendency to the disease underwent a constant increase with the progress of life. Quetelet, on the contrary, concludes that insanity is most frequent between 40 and 50 years of age, and Burrows between 30 and 40.

It is evident that to estimate correctly the relative liability to insanity at different ages, a comparison must be instituted as to the number of *occurring* cases at the different periods of life, and the relations which these bear to the numbers living at the same ages in the general population. The materials which at present exist for instituting a comparison of this kind appear, from our author, to be scanty. The only published data on the subject, extending over a considerable period of time, being prior to those given in the 'Statistics of the Retreat,' a list of 566 cases, by Dr. Jessen, of Schleswig, and the report of Dr. Woodward, of Massachusetts, U.S. Dr. Thurnam, from these and other data, has compiled a table, showing the age at attack in 5122 cases; and a second, from the reports of the Hanwell Asylum for 5 years, 1840-44, from which he infers that the greatest liability to insanity is between the 20th, and 50th and 60th years. The attacks being most common between the 30th and 40th years, and undergoing after this period of life a gradual decrease; a conclusion opposed to that of Esquirol, but harmonizing with the *à priori* inference, that persons at the period of life when the intellectual and physical energies are at their height, and exposure to the influence of disturbing causes the greatest, would be most subject to the disease.

In some of the American asylums, statistical observations show that there is a greater prevalence of the disease between 20 and 30 years of age than at any other period; a fact referrible probably to the peculiar circumstances of the population of that country. The same, from the observations made at the Retreat, would appear to be the case in the Society of Friends. The proportion of cases admitted between 20 and 30 years of age being nearly one third of the whole, and the admissions after this period of life gradually declining.

3. *Liability to insanity in the Society of Friends.* Our author, in entering upon the discussion of this subject, refers to the difficulty of estimating the liability to and prevalence of insanity, either in the general population, or in a particular community like the Society of Friends. In order to determine the liability to disorder in any community, he shows that as in the former instances the calculation must be founded on the number of occurring cases, as a variety of causes affecting the curability of the disease, the tendency to relapses, and the proportion of deaths, will materially affect the number of insane persons living in a given population. From the observations made at the Retreat, it appears that for the 20 years, 1820-40, the number of admissions averaged 12·35 annually; of which 5·45 were males and 6·9 females. Returns recently obtained show also that the mean population of the Society during this period was about 17,900, of whom 8137 were males and 9763 females. From these data it appears that 6·9 cases of insanity are admitted into the Retreat annually out of a population of 10,000 persons. It is further assumed that 2 other cases of insanity occur annually among members of the Society which are not sent to the Retreat, giving a total of 8·9 cases; or, making a deduction of cases readmitted into the Retreat which had not been discharged *cured,* of 8·1 cases out of 10,000 persons, or 1 in

1234. Of the 274 cases received into the Retreat during the twenty years, 163, or nearly two thirds of the whole, viz. 79 males and 84 females, were admitted with first attacks; deducting therefore the cases readmitted, Dr. Thurnam concludes that the actual cases which occur annually in the Society average 5·5 per 10,000 persons, or 1 in 1790.

The cases actually admitted into the Retreat and similarly corrected, amount to 4·55 per 10,000, or 1 in 2196. The males being 4·55, the females 4·3; showing an excess of males, as compared with the relative number of the two sexes in the Society at large, of 13 per cent.

At the asylum of the Society of Friends, at Bloomingdale, near Dublin, the cases admitted are in the proportion of 6·28 annually to a population of 10,000, or 1 in 1590 persons. At the Friends' Asylum, Frankford, U.S., they amount to 7·9 per 10,000, or 1 in 1265 of the population. In these instances it is not known what correction should be made for the cases not sent to the institutions; but the proportion admitted into the Frankford Asylum may perhaps justify the "surmise that mental disorders are somewhat more frequent in the Society of Friends in the United States than in the same community in Great Britain and Ireland."

For these statistics of the prevalence of insanity in the Society of Friends our author claims only an approximative value; they are, however, we may conclude, from the facilities for obtaining information on this subject afforded by the limited population of the Society, more trustworthy than any calculations we possess as to the frequency of the cases in the general population of this country. Till more accurate information in reference to the latter be obtained, the question whether, as supposed by Dr. Burrows, the members of this community be especially subject to attacks of insanity, or whether, as asserted by Dr. Haslam, they enjoy a peculiar immunity from mental diseases, must remain *sub judice*. The infrequency, however, of habits of intemperance among them, which in the population at large forms so fruitful a source of insanity, renders it probable that the prevalence of the disease in the Society of Friends will prove to be below the average in the general population.

The number of existing cases in the Society, as ascertained by the admissions into the Retreat, and corrected for cases not sent to that institution, appears to be in the proportion of 525 to 10,000 of the population, or 1 in 190 persons. This is undoubtedly a high proportion, but it is probably referrible to the favorable hygienic conditions in which the insane of the Society of Friends are placed; and, as suggested by Dr. Thurnam, to slighter cases being placed and continued under treatment than is generally the practice.

### III. HISTORICAL SKETCH AND STATISTICS OF THE RETREAT.

Of the concluding division of Dr. Thurnam's work, space will only allow of our offering a very concise notice. The suggestion for the establishment of an institution by the Society of Friends, to be specially devoted to the reception and treatment of the insane members of that community, emanated from the late William Tuke, who, together with his son Henry Tuke and Lindley Murray, were the most active agents in its formation. To all who are familiar with the state of our asylums for the insane prior to the establishment of the Retreat, and with the influence which the mode of management adopted in this institution has had in the diffusion of more humane methods of treatment, the names of the Tukes, and especially of Samuel Tuke, the present treasurer and author of the 'Description of the Retreat,' published in 1813, will ever be held in esteem.

"By a singular and interesting coincidence," remarks our author, "it was in the spring of 1792, the very year in which the celebrated Pinel commenced the amelioration of the treatment of the insane in France, by the truly courageous act of unchaining nearly fifty supposed incurable and dangerous lunatics at Bicêtre, that the establishment of the Retreat was proposed to the meeting referred to by the late William Tuke. The proceedings of Pinel, however, were not, until long after, known either to the directors or managers of the Retreat; and it thus appears that the reformation in the treatment of the insane had an independent origin in the two countries at the same period."

The institution was first opened for the reception of patients in 1796, and the committee appear to have been singularly fortunate in the choice of its first officers, Dr. Fowler, the visiting physician, and the late George Jepson, the resident superintendent. The testimony to the merits of the latter individual we regret our inability to transcribe in full. "Rarely, we believe, has it happpened that a project so enlightened has met with an agent so efficient for the carrying of it out."

We shall conclude our notice by quoting the report of our author on the experience of the medical officers of the Retreat, on the *quæstio vexata* of this branch of practical medicine—the employment of means of personal restraint:

"The subject of coercion during a refractory, violent, or otherwise dangerous state, or, in other words, the personal restraint of the insane by mechanical means, has of late attracted much attention. At the Retreat, from a very early period, it has been regarded more or less in the light of a necessary evil; and it has been one of the objects of the managers of the institution to resort to it as seldom as possible. The treasurer of the institution, who published his 'Description of the Retreat,' so early as 1813, when detailing the means of personal restraint then employed, observes, 'with regard to the necessity of coercion, I have no hesitation in saying, that it will diminish or increase as the moral treatment of the patient is more or less judicious.' But he immediately adds, 'we cannot, however, anticipate that the most enlightened and ingenious humanity will ever be able entirely to supersede the necessity for personal restraint.'

"In 1841, when the first edition of the 'Statistics of the Retreat' was printed, I made the following observations on this subject:—'Within the last two years the officers of some institutions have attempted, and in some instances apparently with great success, to conduct the management of even large hospitals for insane paupers, without resorting to such means of restraint. The important experiment of this description, if experiment it can now be called, which Dr. Conolly is conducting at Hanwell, must on all hands be regarded with extreme interest, as even if it fail in establishing that personal restraint can in all cases be abolished, it has already fully shown that it may be much more frequently dispensed with, not only with safety, but with advantage to the patient, than has hitherto been generally, if at all, suspected. The officers of the Retreat have not hitherto thought it right, in every case, to dispense with the use of all mild and protecting means of personal restraint; believing that, independently of consideration for the safety of the attendant, they may, in some instances, be regarded as the least irritating, and therefore the kindest method of control. But though this is the case, they readily admit that they have derived advantage from the full consideration of the subject, which the attempts at Hanwell, Lincoln, and elsewhere have induced, and that they remain open to further evidence upon the subject.'

"It is now more than two years since the foregoing was written, and I have the satisfaction of adding that, in practice, personal restraint has by degrees been almost entirely abolished. Whilst we hold ourselves free to direct the use of any means which the necessities of the particular case appear to call for, I am fully convinced that, in a well arranged and properly governed public institution, the instances where personal restraint can at all be considered needful are in truth very few, and that they will be found, almost exclusively, to consist of old or mismanaged cases.

"I have indeed no hesitation in stating what can hardly I think be doubted, that restraint of every description, not absolutely called for, has a tendency to excite in the insane the angry and vindictive passions to which they are only too prone, and thus to prolong the continuance of the disorder in curable cases, and to aggravate its character in incurable ones. The instructions to the attendants on this subject have already been given; and during the current year there has only been a single example of the application of personal restraint, and that was of the mildest possible kind. There has been no instance of its application since the 25th of January, 1843."

In a note at p. 105 of the first part, we are further informed that in 4 cases slight personal restraint had been had recourse to between the 25th of January, 1843, and the 8th of February, 1845—and, in a letter recently published in another periodical, it is stated that no instance of its employment in any form has since occurred; so that the use of coercion in the institution may be regarded as virtually abolished. Dr. Thurnam adds—

"Whilst on the one hand I cannot doubt that the course and duration of many cases have been mitigated and shortened, and the character of the disorder rendered less virulent, by the disuse of restraint, I must here state that occasional inconvenience in the shape of alarm, and of interruption to the quiet of other patients, and also as regards the destruction of clothing and the breakage of glass, have, on the other hand, been connected with it. On the whole, however, that greater vigilance and forbearance on the part of the attendants, which under competent superintendence the comparative disuse of personal restraint more or less necessarily implies, have, I feel no hesitation in saying, been attended with a decided increase of comfort and decrease of irritation in those divisions of the establishment in which instruments of restraint were formerly not unfrequently resorted to."

We must now take our leave of Dr. Thurnam's volume, omitting, from want of space, any notice of the concluding portion—the 'Statistics of the Retreat.' The value which our readers will attach to this work, will probably much depend on their views as to the importance of statistical investigation in general. For our own part, though fully alive to the cautions necessary in the application of numbers to the elucidations of questions of medical science, and to the fallacious conclusions which may be drawn from hasty, inaccurate, or too scanty observations, we believe that there are numerous points regarding the natural history of diseases, and the various causes which may modify their symptoms, progress, and results, and the effects of remedial agents, which can only be satisfactorily solved by the employment of the numerical method. This we think peculiarly the case as regards insanity, and we may appeal to Dr. Thurnam's work as an example of the interesting and novel results which may thus be deduced; and which, though they might be suggested by ordinary individual experience, are only capable of being established as facts, by calculations founded on a large number of observations. Those only who, like ourselves, have been somewhat extensively engaged in statistical researches, will be able fully to appreciate the amount of labour and care which, to ensure the accuracy which throughout characterises Dr. Thurnam's work, must have been required in its compilation; and we cannot but regret that, by adopting a more simple and lucid arrangement, and condensing his matter into a smaller space, he had not presented his observations in a more attractive form. To the medical officers of hospitals for the insane, and indeed to all those engaged in the cultivation of medical statistics, his work will afford much valuable information as to the methods to be adopted in the numerical registration of the results of their experience, and the errors to be avoided in generalizing from them. To such we believe the abstract we have given will be a sufficient recommendation. To the general professional reader, works of this kind must necessarily be less attractive than those of a more practical description, and we have hence been the more anxious to present as full an analysis of it as our limits would permit. We feel convinced, however, that those who are engaged in the cultivation of this branch of practical medicine will find sufficient in the work itself to reward them for its perusal.

---

